# Evaluating the impact of incorporating clinical practice guidelines for the management of infectious diseases into an electronic application (e-app)

**DOI:** 10.1017/ice.2022.286

**Published:** 2023-09

**Authors:** Holly M. MacKinnon, Kathryn L. Slayter, Jeannette L. Comeau, Caroline King, Emily K. Black

**Affiliations:** 1 Dalhousie University, Halifax, Canada; 2 IWK Health, Halifax, Canada; 3 Lawton’s Drugs, Halifax, Canada; 4 Nova Scotia Health, Halifax, Canada

## Abstract

**Objectives:**

To improve dissemination and accessibility of guidelines to healthcare providers at our institution, guidance for infectious syndromes was incorporated into an electronic application (e-app). The objective of this study was to compare empiric antimicrobial prescribing before and after implementation of the e-app.

**Design:**

This study was a before-and-after trial.

**Setting:**

A tertiary-care, public hospital in Halifax, Canada.

**Participants:**

This study included pediatric patients admitted to hospital who were empirically prescribed an antibiotic for an infectious syndrome listed in the e-app.

**Methods:**

Data were collected from medical records. Prescribing was independently assessed considering patient-specific characteristics using a standardized checklist by 2 members of the research team. Assessments of antimicrobial prescribing were compared, and discrepancies were resolved through discussion. Empiric antimicrobial prescribing before and after implementation of the e-app was compared using interrupted time-series analysis.

**Results:**

In total, 237 patients were included in the preimplementation arm and 243 patients were included in the postimplementation arm. Pneumonia (23.8%), appendicitis (19.2%), and sepsis (15.2%) were the most common indications for antimicrobial use. Empiric antimicrobial use was considered optimal in 195 (81.9%) of 238 patients before implementation compared to 226 (93.0%) 243 patients after implementation. An immediate 15.5% improvement (*P* = .019) in optimal antimicrobial prescribing was observed following the implementation of the e-app.

**Conclusions:**

Empiric antimicrobial prescribing for pediatric patients with infectious syndromes improved after implementation of an e-app for dissemination of clinical practice guidelines. The use of e-apps may also be an effective strategy to improve antimicrobial use in other patient populations.

Antimicrobial resistance (AMR) is a threat to the health of the population worldwide. According to the World Health Organization, resistance of commonly encountered bacteria has spread to all regions of the world.^1^ Recognizing the threat to Canadians, the government of Canada aims to “strengthen the promotion of the appropriate use of antimicrobials.”^2^

Clinical practice guidelines are a strategy to encourage prescribing using best practices. Although guidelines have the potential to improve patient care, nonadherence has been reported.^3–5^ Potential reasons for nonadherence include lack of awareness and/or familiarity of guidelines, which may occur due to the volume of information provided, time to stay informed and updated with existing guidelines due to workload constraints, and guideline accessibility.^6^ A potential solution to improve accessibility includes incorporation of guidelines into an electronic application (e-app) available to healthcare providers on mobile devices.

From December 2015 to March 2017, the IWK Health antimicrobial stewardship program (ASP) in consultation with clinical divisions, created the IWK antimicrobial stewardship (AMS) e-app. The e-app included the following 3 sections: (1) syndromes, (2) antimicrobials, and (3) pathogens.

1. Syndromes: Evidence-based empiric treatment guidelines were included for 55 pediatric infectious syndromes. Under each syndrome, the e-app listed likely pathogens, provided suggested antimicrobial regimens that included drug, dose, route of administration, and duration of therapy to treat the syndrome, and duration of therapy.
2. Antimicrobials: The e-app included spectrum of activity, dosing regimens, drug monitoring, common usage, adverse effects, drug interactions, risk of *Clostridioides difficile*, oral bioavailability, estimated cost/day, and pharmacology (antimicrobial class, route of elimination, and average serum half-life).
3. Pathogens: The pathogen section included information on infection prevention and control precautions, local susceptibilities (percentage of isolates susceptible to specific antimicrobial agents), associated syndromes, and epidemiology.

The iPhone- and Android-compatible e-app was launched in May 2017. In the first 24 hours after the e-app was released, the e-app was downloaded 157 times and was accessed 1,193 times. A year after the e-app was implemented, it continued to be downloaded and accessed; on average, >700 users accessed the e-app 1,500–2,000 times per month. A recently completed local qualitative study of antimicrobial use and stewardship by our team also identified use of the e-app as a facilitator to improving antimicrobial use.^7^ Although the e-app was frequently accessed and perceived as helpful by healthcare providers, the impact of this e-app on antimicrobial prescribing at our institution is unknown. To our knowledge, the impact of an e-app on antimicrobial prescribing for pediatric patients has not been published elsewhere in the literature.

In this study, we evaluated the impact of incorporating locally developed guidelines for management of infectious syndromes into an electronic e-app on empiric antimicrobial prescribing in pediatric patients.

## Methods

### Study design

An interrupted time-series (ITS) design was used for this study. In an ITS study, measurements of the outcome are taken repeatedly at equal intervals (monthly in this investigation) both before and after the intervention. The underlying trend of the outcome can then be established and accounted for in the final model, making this study design less susceptible to certain biases including secular trends and seasonal variations in drug use.^8^ The key assumption is that the trend before implementation represents what would have happened in the postintervention period had the policy not been implemented. Because this cannot be directly verified using the data, substantive knowledge regarding whether there are other changes in the postimplementation period that could plausibly have affected the outcome need to be assessed. To the best of our knowledge, no other major changes (administrative or clinical) occurred during our 1-year follow-up period that were likely to affect our outcome of optimal antimicrobial prescribing.

### Setting and study population

This study was completed at IWK Health in Halifax, Canada. The IWK is a 271-bed tertiary-care center serving the Maritime Provinces in eastern Canada. Pediatric patients aged 0–16 years admitted to the IWK Health who were diagnosed with 1 of the 55 infectious syndromes detailed in the e-app and were prescribed a systemic antimicrobial agent were considered for inclusion. A list of infectious syndromes included in the e-app at the time of data collection is outlined in the Supplementary Materials (online). Only the first instance of an infection meeting inclusion criteria for each patient encounter was included. Patients transferred to IWK Health from other institutions who were already on antimicrobial agents were excluded.

### Data collection

Using *International Classification of Disease, Tenth Revision* (ICD-10) codes, decision support at IWK Health identified patients diagnosed with one of the infectious syndromes listed in the e-app. A research assistant reviewed electronic charts through our local electronic medical record (EMR), Meditech, and extracted data on a random sample of patients (target of 5 patients per week or ∼45%–50% of all qualifying patient encounters) meeting inclusion criteria 1 year before (May 1, 2016, through April 30, 2017) and 1 year after implementation (May 15, 2017, through May 15, 2018) of the e-app using a standardized data collection tool. Patients were randomly selected from a monthly list of admissions using a random-number generator. Extracted data included the following: patient demographics (age, sex, weight, date of admission), infectious diagnoses (including date and time of diagnosis), microbiological culture and sensitivity results, radiology findings related to the infection (if applicable), comorbidities, organ function, allergy status, empiric and targeted antimicrobial use (including date and time prescribed and/or administered), and other concomitant medications. Data on patient outcomes including mortality and length of hospital were also collected.

Empiric antimicrobial prescribing was then objectively evaluated using a previously developed set of quality indicators. These quality indicators were developed by our research team using the Delphi technique and have been described previously.^9^ A panel of 12 experts (pediatric pharmacists and infectious diseases physicians) rated indicators and had the opportunity to suggest additional indicators using this technique. Only indicators that achieved consensus on empiric choice of antimicrobial prescribing after 3 rounds were used to evaluate prescribing in this study. Indicators we used to assess for appropriate empiric choice of antimicrobial therapy considered adherence to local guidelines, local antibiogram, and patient specific factors including previous history of infection, recent travel, antibiotic allergy, and underlying comorbidities. Our team did not assess appropriateness of dose, route of administration, or duration of antimicrobial therapy. Two members of the research team, who are pharmacists with postgraduate doctorate degrees and experience in pediatrics and infectious diseases (K.S. and E.B.), independently evaluated choice of empiric antimicrobial prescribing using these indicators while considering patient-specific factors. Reviewers had access to electronic records to clarify data collection if required and as a result were not blinded. All disagreements were resolved through discussion.

The primary outcome of interest in this study was optimal empiric choice of antimicrobial prescribing. Secondary outcomes of interest included mortality and length of hospital stay.

### Descriptive analyses

Demographic and antimicrobial data were analyzed descriptively using means (with standard deviations) and proportions (Table 1). These are reported for the full cohort, the preimplementation cohort and the postimplementation cohort. To understand how the population might be changing over time, the means and proportions between the preimplementation and postimplementation cohorts were compared using *t* tests and χ^2^ tests, respectively.

**Table 1.** Baseline Characteristics of Pediatric Patients With a Suspected or Confirmed Infection

Characteristic	Total (N = 480), No. (%)	Before the Intervention (N = 237), No. (%)	After the Intervention (N = 243), No.(%)	*P* Value
**Age**				<.001
<28 d	49 (10.2)	36 (15.2)	13 (5.4)	
29 d–1 y	72 (15.0)	41 (17.3)	31 (12.8)	
>1 y	359 (74.8)	160 (67.5)	199 (81.9)	
Age, mean y	5.5 (4.9)	4.9 (4.9)	6.1 (4.8)	.007
Sex, male	55.32%	55.51%	55.14%	.94
**Indication**				.008
Pneumonia	114 (23.8)	50 (21.1)	64 (26.4)	
Appendicitis	92 (19.2)	40 (16.9)	52 (21.4)	
Sepsis	73 (15.2)	51 (21.5)	22 (9.1)	
Urinary tract infection	63 (13.1)	34 (14.4)	29 (11.9)	
Infections of the head and neck	59 (12.3)	26 (11.0)	33 (13.6)	
Skin and soft-tissue infections	27 (5.6)	14 (5.9)	13 (5.4)	
Other	52 (8.3)	22 (9.3)	30 (12.3)	
Optimal empiric antimicrobial prescribing	420 (87.5)	194 (81.9)	226 (93.0)	<.001

### ITS analyses

For our ITS model, we hypothesized that the e-app could have both an immediate impact (referred to as a level change) and a gradual change in the gradient of the outcome trend. We used a segmented linear regression model. Our outcome was modeled as the proportion of prescriptions that were optimally prescribed in each month. Four regression parameters were estimated in this model: (1) the baseline level when time is zero (ie, *y*-intercept), (2) the underlying preintervention trend, (3) the level change following the intervention, and (4) the slope change following the intervention. All analyses were conducted using Stata version 16 software (StataCorp, College Station, TX). The Stata package *itsa* was used to create the ITS model estimates and graphics.^10^

## Results

**Fig. 1.**
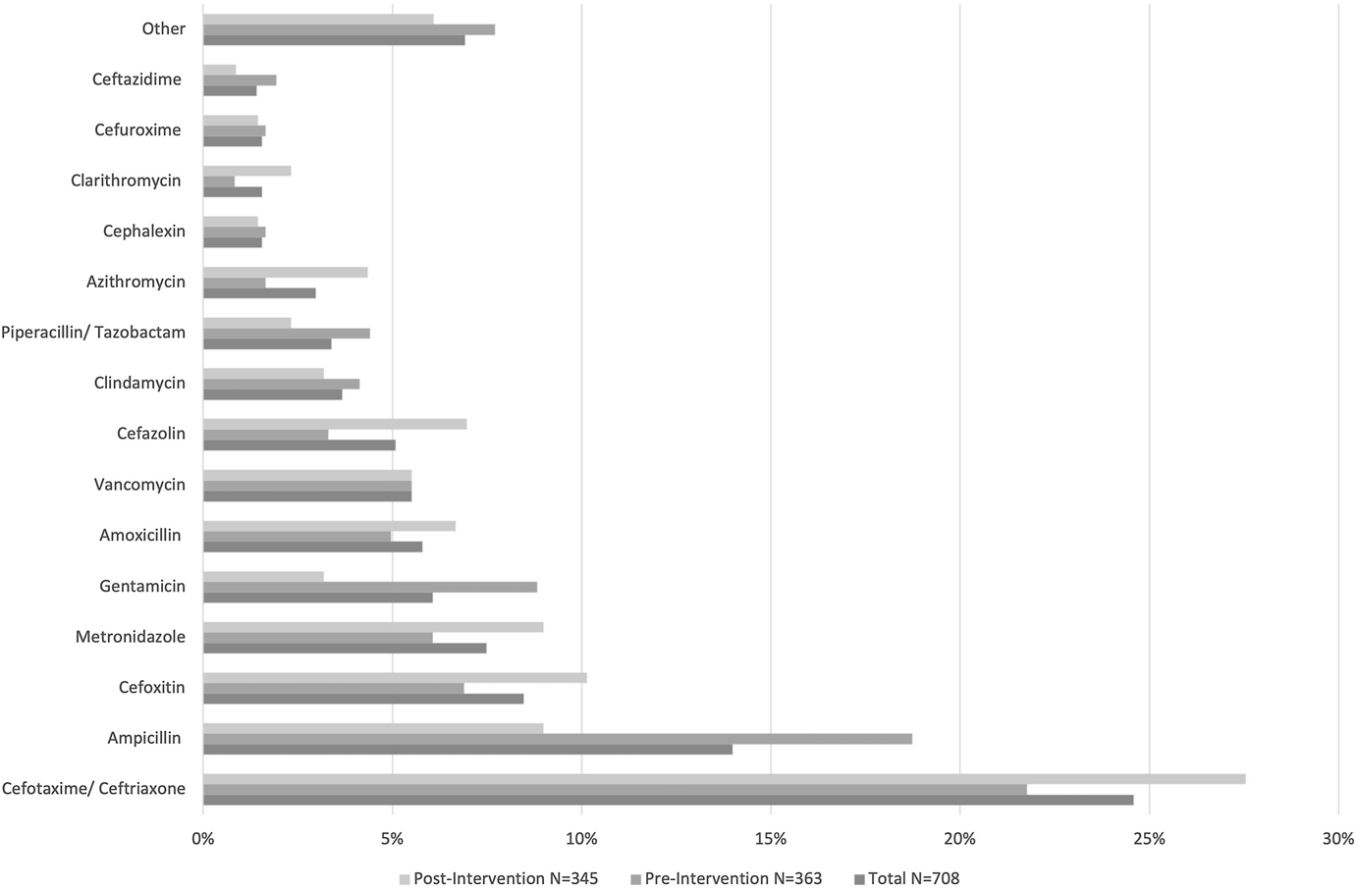
Empiric antimicrobials prescribed to pediatric patients with confirmed or suspected infections.

In total, 237 of a possible 453 patient encounters were included in the preintervention period, and 243 of a possible 554 patient encounters were included in the postintervention period. The mean age of our patient population was 5.5 years. Overall, the most common indication for empiric antimicrobial prescribing was pneumonia (23.8%). In total, 708 empiric antimicrobials were prescribed for 480 patient encounters in our study and the most common antimicrobials prescribed were ceftriaxone or cefotaxime. Baseline characteristics of our patient population are outlined in Table 1. A descriptive summary of empiric antimicrobials prescribed is outlined in Figure 1.

Empiric antimicrobial prescribing demonstrated a significant immediate improvement after implementation of the e-app. As shown in the regression table (Table 2), the starting level of appropriate empirical antimicrobial prescribing was estimated at 85.3%, and appropriateness appeared to decrease slightly at −0.8% per year during the preintervention period although this was not statistically significant (95% CI, −2.1 to 0.5; *P* = .20). In the first month of the intervention, May 2017, we detected a significant increase in appropriate prescribing of 15.5% (95% CI, 2.8–28.3; *P* = .02), followed by a 1.2% improvement per month relative to the preintervention trend, but this trend was statistically insignificant (95% CI, −0.2 to 2.68; *P* = .10). The combined results suggest that the e-app had a large, positive, immediate impact that was sustained over the following year.

**Table 2.** Estimates From Interrupted Time-Series Analysis Using a Segmented Linear Regression Model

Characteristics of Prescription Percentages That Were Optimally Prescribed	Coefficient	95% Confidence Interval	*P* Value
Intercept (baseline when time is zero)	85.3	78.2–92.5	.00
Preintervention trend	−0.8	−2.1 to 0.5	.20
Level change	15.5	2.8–28.3	.02
Slope change after the intervention	1.2	−0.2 to 2.6	.10

**Fig. 2.**
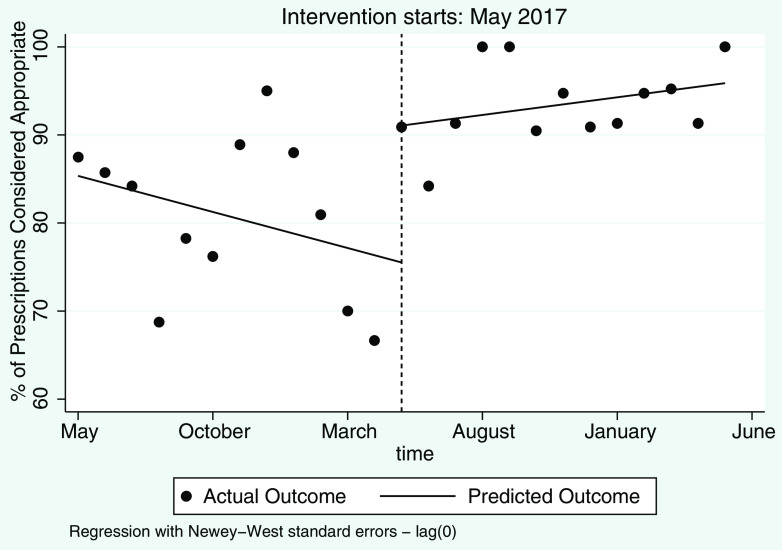
Empiric antimicrobial prescribing for pediatric patients with suspected or confirmed infections. Dots represent monthly averages, and the lines represent the predicted values from the segmented regression model.

Additionally, as can be seen in Figure 2, there appears to be less variation in the outcome after the implementation of the e-app, suggesting that the e-app not only improved optimal prescribing but also consistency. We tested for homogeneity of variances between the preimplementation and postimplementation periods using the Levene robust test statistic (Table 3). We obtained *P* < .0001, which indicates at an α level of 0.05 that we can reject the null hypothesis that the variances are equal. Thus, we did detect a statistically significant difference in the variances between the preimplementation and postimplementation period. Additionally, although these trends were not compared statistically due to small sample size, a similar trend in improvement was observed in empiric choice of antimicrobial agents after the intervention for the most common indications (Table 4).

**Table 3.** Testing for Difference in Variances for Mean Prescriptions That Were Optimally Prescribed Before and After Variation Using the Levene Test Statistic

Period	Prescriptions That Were Optimally Prescribed, %	Standard Deviation	Levene *P* Value
Before the intervention	81.8	38.6	
After the intervention	93.0	25.6	<.001

**Table 4.** Rate of Optimal Antimicrobial Prescribing by Indication Before the Intervention Versus After the Intervention for Most Common Indications

Indication	Before the Intervention, No. (%)	After the Intervention, No. (%)
Pneumonia	50/64 (78.1)	86/92 (93.5)
Appendicitis	46/62 (74.2)	68/68 (100.0)
Sepsis	97/107 (90.7)	45/45 (100.0)
Urinary tract infections	40/49 (81.6)	32/34 (94.1)
Infections of the head and neck	20/32 (62.5)	37/45 (82.2)

The mortality rates before the intervention compared to the postintervention period have not been reported due to the low rate of deaths reported (N < 5). Length of stay was also compared using an ITS analysis, but there was no significant change in this outcome.

## Discussion

An immediate and sustained improvement in empiric antimicrobial prescribing was observed in our study after implementation of the e-app. This study is the first to evaluate implementation of an e-app to disseminate guidelines for prescribing of antimicrobials for pediatric inpatients. Our results add to literature on use of smartphone apps to improve prescribing of antimicrobial agents by evaluating impact of the e-app in the pediatric population using an ITS study design.

Evidence evaluating implementation of smartphone apps have been published in other populations and for specific indications. Charani et al^11^ published a study that evaluated use of a smartphone decision support tool over a 6-year period using an ITS study design. Consistent with findings from our ITS analysis, Charani et al found a significant immediate improvement of 6.63% in choice of antimicrobial after implementing the intervention on surgical units. Improvement was observed in medicine patients as well; however, this did not reach statistical significance. A lack of significant improvement may have occurred due to high baseline adherence to local antimicrobial policies, which exceeded 80% for most time points prior to implementation of the prescribing tool.^11^ Similarly, our study had a high baseline rate of adherence; however, unlike Charani et al, we were able to demonstrate significant benefit after implementation of the e-app in our patient population.

A systematic review of 13 studies that evaluated smartphone apps for prescribing antimicrobials in hospitals was also completed by Helou et al^12^ (including the study by Charani et al^11^); they primarily evaluated process indicators and user experience. Most studies in this review were before-and-after or cross-sectional in design, with low to moderate quality. Many of these studies solely assessed user experience. Only 4 studies included in this review evaluated adherence to guidelines. Like results from our study, evaluated studies included in the systematic review (N = 4) found improved prescribing with implementation of an e-app. Definition of appropriate therapy varied by study and Helou et al^12^ highlighted the need for future research to clearly defined quality indicators for defining appropriateness. These researchers concluded that evidence on use of these e-apps was limited, primarily focused on a small number of indications, and that additional high-quality studies were needed.^12^

Our study had several strengths that address limitations reported elsewhere. The ITS design we used in this study is a quasi-experimental design ideally suited for real-world interventions with clearly defined pre- and postintervention periods. To our knowledge, no other interventions were implemented during the study period that might have had a significant impact on empiric antimicrobial prescribing suggesting a direct impact of the e-app. Furthermore, 2 members of our research team independently assessed choice of empiric prescribing using a standardized checklist of quality indicators for appropriate empiric prescribing in pediatric patients that was developed and achieved consensus from a panel of experts.^9^ In addition, we assessed prescribing in a wide variety of indications to understand overall impact of the e-app on antimicrobial use at our institution.

Despite these strengths, several limitations should be considered. Our assumptions that the preintervention mean is linear and that the characteristics of the population remain unchanged throughout the study period are unlikely to be fully met here. Given our relatively limited sample size, we wanted to be cautious to not ‘overinterpret’ the preintervention data; therefore, we did not attempt to add additional parameters (eg, quadratic terms or seasonality). The variation in the preintervention data is reflected in the wide confidence intervals, particularly in our level-change estimate. Despite this potential limitation, the consistent optimal prescribing after implementation of the e-app suggests benefit of the intervention. Regarding the characteristics of the population remaining unchanged throughout the study period, a younger patient population (<28 days) and more infants with sepsis were observed prior to implementation of the e-app, whereas an older pediatric population of patients with pneumonia and appendicitis was observed in the postimplementation phase. Although these differences may have affected findings, it is likely this variability resulted in more favorable prescribing in the preintervention phase given that a higher proportion of these patients had sepsis. Prescribing was deemed optimal in most patients with sepsis (>90%) in both the preintervention and postintervention cohorts.

Furthermore, to allow reviewers assessing appropriateness to access patient charts if required for clarification, reviewers were not blinded. To minimize this bias, we used a standardized list of indicators when assessing appropriateness; however, some subjectivity remains. In addition, we identified patients using ICD codes. Miscoding by data analysts is possible using ICD codes and may have resulted in misclassification.

Finally, our study may not be generalizable to other settings. Additional research is needed to explore the potential benefit of an e-app in other settings, including primary care. Finally, we only assessed empiric choice of antimicrobial therapy. Further research is needed to explore the impacts on other quality indicators of appropriate antimicrobial prescribing such as dose, route of administration, and duration of antimicrobial use.

Despite these limitations, our study adds evidence to the literature and suggests that implementation of an e-app for dissemination of pediatric guidelines may result in improved choice of empiric antimicrobial for patients with suspected or confirmed infections. The results of this study may be considered by other institutions considering incorporation of their institutional guidelines into an e-app to improve uptake and adherence to best practice. The most significant impact of implementing an e-app may be observed when targeting inclusion of specific indications where the greatest variability in prescribing practices is identified at baseline. Findings from our study concur with the results of previous qualitative work our team has completed, highlighting the value of implementing an e-app^7^ and suggesting an immediate benefit and a more consistent, appropriate choice of empiric antimicrobial prescribing after implementing an e-app.

## Supplementary material

For supplementary material accompanying this paper visit https://doi.org/10.1017/ice.2022.286. click here to view supplementary material
