# Supplementary material for: Evaluating the impact of incorporating clinical practice guidelines for the management of infectious diseases into an electronic application (e-app)
Source: Infect Control Hosp Epidemiol. 2023 Jan 3;44(9):1417–22. doi: 10.1017/ice.2022.286 (PMC10507496; doi:10.1017/ice.2022.286)
Supplement: Supplementary file 1 [file S0899823X22002860sup001.docx]

**Supplementary Table – Infectious Syndromes included in the IWK Health App**

| **Infective Endocarditis** |
| --- |
| **Central Nervous System**  Cerebral abscess or subdural empyema  Meningitis  Encephalitis  Neurological Lyme (isolated facial palsy,  meningitis, encephalitis) |
| **Genito-urinary Tract**  Pelvic inflammatory disease  Urinary tract infection |
| **Head and Neck**  Acute otitis media  Periorbital or Orbital Cellulitis  Cervical lymphadenitis (Acute, Subacute,  atypical mycobacterial)  Dacrocystitis  Epiglottitis / tracheitis  Mastoiditis  Retro/Para Pharyngeal Cellulitis/Abscess  Sinusitis  Streptococcal pharyngitis / tonsillitis  Tooth infections & dental abscess, including pulpitis |
| **Intra-abdominal/Gastrointestinal**  Antibiotic-associated colitis (Clostridium  Difficile Infection)  Appendicitis  Cholangitis/Cholecystitis  Gastroenteritis  Pinworms  Primary peritonitis |
| **Lower Respiratory Tract**  Aspiration Pneumonia  Community-acquired Pneumonia  Parapneumonic effusion/empyema  Nosocomial acquired pneumonia |
| **Neonatal Intensive Care Unit (NICU)**  Early Onset Sepsis (EOS)  Late Onset Sepsis (LOS)  Necrotizing Enterocolitis (NEC), confirmed |
| **Osteoarticular**  Lyme Arthritis  Osteomyelitis  Septic Arthritis |
| **Pertussis** |
| **Sepsis** |
| **Skin and Soft Tissue**  Cellulitis  Dog / cat / human bites  Herpes Simplex Virus neonates: Localized SEM (skin, eye, mouth)  Lyme Erythema Migrans  Necrotizing Fasciitis  Periorbital / Orbital Cellulitis |
